# Evaluation of the annual Canadian biodosimetry network intercomparisons

**DOI:** 10.3109/09553002.2015.1012305

**Published:** 2015-02-27

**Authors:** Ruth C. Wilkins, Lindsay A. Beaton-Green, Sylvie Lachapelle, Barbara C. Kutzner, Catherine Ferrarotto, Vinita Chauhan, Leonora Marro, Gordon K. Livingston, Hillary Boulay Greene, Farrah N. Flegal

**Affiliations:** ^a^ Health Canada, Environmental Radiation and Health Sciences Directorate, Ottawa, ON, Canada; ^b^ Oak Ridge Associated Universities, REAC/TS, Radiation Emergency Medicine (REM), Oak Ridge, TN, USA; ^c^ Radiological &amp; Nuclear Defence and Navigation Warfare, Defence R&amp;D Canada-Ottawa Research Centre, Ottawa, ON; ^d^ Canadian Nuclear Laboratories Limited, Chalk River, ON, Canada

**Keywords:** Intercomparison, biodosimetry, dicentric chromosome assay, emergency response

## Abstract

*Purpose*: To evaluate the importance of annual intercomparisons for maintaining the capacity and capabilities of a well-established biodosimetry network in conjunction with assessing efficient and effective analysis methods for emergency response.

*Materials and methods*: Annual intercomparisons were conducted between laboratories in the Canadian National Biological Dosimetry Response Plan. Intercomparisons were performed over a six-year period and comprised of the shipment of 10&ndash;12 irradiated, blinded blood samples for analysis by each of the participating laboratories. Dose estimates were determined by each laboratory using the dicentric chromosome assay (conventional and QuickScan scoring) and where possible the cytokinesis block micronucleus (CBMN) assay. Dose estimates were returned to the lead laboratory for evaluation and comparison.

*Results*: Individual laboratories performed comparably from year to year with only slight fluctuations in performance. Dose estimates using the dicentric chromosome assay were accurate about 80% of the time and the QuickScan method for scoring the dicentric chromosome assay was proven to reduce the time of analysis without having a significant effect on the dose estimates. Although analysis with the CBMN assay was comparable to QuickScan scoring with respect to speed, the accuracy of the dose estimates was greatly reduced.

*Conclusions*: Annual intercomparisons are necessary to maintain a network of laboratories for emergency response biodosimetry as they evoke confidence in their capabilities.

## Introduction

Biological dosimetry has been employed for many years as a method for estimating the dose of ionizing radiation received by an individual. This information is critical to the medical community as it assists with effective and timely treatment regimens for potentially exposed individuals (Waselenko et al. 2004, International Atomic Energy Agency [IAEA] 2011, Sullivan et al. 2013), or for identifying radiation workers who are near or have exceeded their limit for exposure.

To date, several biological markers have been developed to measure radiation-induced damage. Traditionally, the dicentric chromosome assay (DCA), which provides dose estimates based on the frequency of dicentric chromosomes in peripheral blood lymphocytes, has been the method of choice. The DCA is very sensitive due to a low and stable background dicentric frequency (0.5&ndash;1 per 1000 metaphase spreads) (IAEA 2011) and is specific to damage from ionizing radiation. Using this assay, dose levels as low as 0.1&ndash;0.2 Gy can be detected when 500&ndash;1000 metaphase spreads are analyzed, but this requires many hours of analysis (IAEA 2001). In a mass casualty event, however, where medical treatment would be administered only to those receiving more than 2.0 Gy, this level of sensitivity is not required (Sullivan et al. 2013). In these situations, the sensitivity of the assay can be reduced by decreasing the number of metaphase cells scored which subsequently greatly reduces the time required for analysis. Standard triage DCA analysis now consists of analyzing only 50 metaphase spreads, providing a threshold of detection of 1&ndash;2 Gy; still adequate to guide treatment of acute radiation syndrome (ARS) (Lloyd 1997, Lloyd et al. 2000, Voisin et al. 2001, International Organization for Standardization [ISO] 2008).

The time efficiency of triage-based scoring has been further improved dramatically, without losing accuracy in the dose estimate, by introducing a scoring technique termed &lsquo;DCA QuickScan&rsquo; (Flegal et al. 2010, 2012). The basis for this method is that individual centromeres are not counted and metaphase spreads are only rapidly examined for obvious damage, thereby eliminating the counting of individual chromosomes to ensure the completeness of the analyzed cell, as done in the conventional DCA (CDCA) method. This method has been demonstrated to be as accurate as conventional triage scoring, while reducing the time for scoring by a factor of about 6 (Flegal et al. 2012).

The cytokinesis block micronucleus (CBMN) assay has also been demonstrated to be a useful tool for biological dosimetry. In this assay, the frequency of micronuclei (MN) in binucleated cells (BNC) is used as a measure of damage from ionizing radiation. Although these MN are not radiation specific, they do increase with dose and have been validated as a technique for the estimation of exposures to radiation (Fenech et al. 2003, Vral et al. 2011). The advantage of this method over the DCA is that the manual scoring is much faster, as only 200 BNC are required to provide a sensitivity of 1 Gy (McNamee et al. 2009), and the method requires less technical expertise.

Another strategy for increasing the throughput of biological dosimetry is the development of biodosimetry laboratory networks. Several networks have already been established to improve dose estimation throughput, such as the National Biological Dosimetry Response Plan (NBDRP) in Canada (Miller et al. 2007), the Latin American Biological Dosimetry Network (Garcia et al. 1995) and the Chromosome Network in Japan (Yoshida et al. 2007). In addition and the European Network, Realizing the European Network of Biodosimetry (RENEB) is on its way to being established (Kulka et al. 2012). When a network is established, it is imperative to perform regular intercomparisons between the laboratories of the network to maintain and assess accuracy and throughput. Many one-time intercomparisons have been conducted over the past few years, both between laboratories within a network (Garcia et al. 1995) and between laboratories from different networks or countries (Roy et al. 2004, Wilkins et al. 2008, Di et al. 2011, Beinke et al. 2013) each with different designs. This paper aims to describe the results of intercomparisons held by the National Biodosimetry Response Plan (NBDRP) in Canada over the past 6 years. Exercises of similar design were conducted each year which included the DCA, both conventional and QuickScan and the CBMN assay. These exercises involved each of the four Canadian reference laboratories as well as the occasional participation of two biodosimetry laboratories from the United States. The lessons learned from these intercomparisons will be discussed with an emphasis on demonstrating the importance of repeated intercomparison exercises.

## Methods

### Blood collection, irradiation and transportation

All blood donors were volunteers who willingly responded to an advertising call for participation in a research protocol approved by Health Canada Research Ethics Board. All donors gave informed consent and none had a recent history of ionizing radiation exposure. For each exercise, blood samples were drawn from each of 10&ndash;12 donors (ages 20&ndash;60 years), by venipuncture into 4 ml lithium heparinized Vacutainer^&reg;^ tubes (Becton Dickinson, Oakville, ON, Canada). Irradiation of all blood samples was completed *ex vivo* in the Vacutainer^&reg;^ collection tubes at room temperature. The irradiation system used varied from year to year based on availability as outlined in Table I.

**Table I.** Irradiation conditions for each intercomparison.

Year	Radiation quality	Dose rate (Gy/min)	Unit	Calibration
2007	^137^Cs	0.83	Gammacell 40 (Atomic Energy of Canada Ltd, Ottawa, ON)	Fricke Dosimetry
2008	^137^Cs	0.81	Gammacell 40	Fricke Dosimetry
2009	250 kV X-rays, 12.5 mA, 2mm Al filtration	0.92	XRAD-320 (Precision X-ray Inc. North Branford, CT)	Radcal 9010 ion chamber (Radcal, Monrovia, CA)
2010	250 kV X-rays, 12.5 mA, 2mm Al filtration	1.7	XRAD-320 (Precision X-ray Inc.)	Radcal 9010 ion chamber
2011	^60^Co	0.293	JL Shepherd and Associates San Fernanado, CA	Optically stimulated luminescence dosimetry (OSL)
2012	^60^Co	0.596	JL Shepherd and Associates	OSL

Irradiations at 10&ndash;12 different dose points between 0.0 and 5.0 Gy were delivered to each set of samples, respectively, such that each laboratory received matched irradiated samples from the same donors. Immediately after irradiation, samples were incubated for 2 h at 37&deg;C to allow for repair and blinded bar coded samples from each dose-point were sent to each of the participating laboratories: Health Canada (HC), Defence Research and Development Canada-Ottawa Research Centre (DRDC), Canadian Nuclear Laboratories Limited (CNL), McMaster University, Oak Ridge Institute for Science Education (ORISE) and the Armed Forces Radiobiology Research Institute (AFRRI). The laboratories were informed of the radiation quality prior to the intercomparison. Samples were dispatched either by air with FedEx (AFRRI, ORISE, McMaster) or transported by road (DRDC, CNL). Shipment procedures simulated those during an actual event and followed the Canadian Transportation of Dangerous Goods Regulations for Class 6.2, UN3373 and labelling for Biological Substances Category B. Specifically, the Vacutainer^&reg;^ tubes were shipped according to packaging instructions 650 of International Air Transportation Association (IATA). The package was prominently labeled to indicate that it should not be frozen or X-rayed at airport security checkpoints. As a quality control measure, the package included a temperature data logger for monitoring temperature, which showed that the samples remained between 14 and 27&deg;C during transit and an optically stimulated luminescence (OSL) dosimeter to rule out X-ray screening at airports.

### Cell culture and harvest

Although sample processing protocols varied slightly from laboratory to laboratory, for both the DCA and CBMN assays, they were based on the general guidance provided by the IAEA (IAEA 2001, 2011) and ISO 19238 and 21243 (ISO 2004, 2008).

### Dicentric chromosome assay

Each laboratory set up a whole blood or isolated lymphocyte culture for harvesting metaphase spreads. The cells were added to culture medium (RPMI 1640) containing 15% fetal bovine serum, with L-glutamine, penicillin, streptomycin and 15 &mu;M BrdU and stimulated to cycle by the addition of 2% phytohemagglutinin (PHA). The cells were incubated at 37&deg;C and 5% CO_2_ for 48 h, mitotic arrest was done using 1% colcemid at 10 mg/ml at 44 h (for HC) and 48 h (for DRDC) and then harvested for metaphase spreads to determine the yield of radiation-induced dicentrics. Only first-division metaphase spreads were used for counting dicentrics. The standard method used to ensure that only first-division metaphase spreads were scored was based on either fluorescence-plus-Giemsa (FPG) staining technique coupled with BrdU and Hoechst 33258 or on the addition of cytochalasin B to the cultures after 24 h to inhibit cytokinesis. The FPG staining allows identification by the differential staining of second-division metaphase spreads. The metaphase spreads were harvested after a brief treatment in a suitable hypotonic solution such as 0.56% (0.075 mM) potassium chloride and fixation in 3:1 methanol: glacial acetic acid Carnoy&apos;s fixative. A temperature and humidity-controlled chamber was used to prepare metaphase spreads on glass slides. Multiple slides were prepared for each dose and stained with Giemsa or FPG for chromosome aberration analysis using brightfield microscopy.

In each laboratory, sufficient slides were prepared such that each scorer could score up to 50 metaphase cells or 30 dicentrics on one slide. Conventional DCA scoring followed the recommendations of ISO (ISO 2004) and IAEA (IAEA 2011), and the method for QuickScan scoring was as described by Flegal et al. (2012) except without the stipulation that examination stopped if five dicentrics were seen in less than 20 metaphases.

### Cytokinesis block micronucleus assay

Cell culture was performed similarly to the DCA except that no colcemid was added. Instead cytochylasin B was added after 48 h of incubation to inhibit cytokinesis, and cells were cultured for a total of 72 h. Cells were first fixed with 5:1 methanol: glacial acetic acid and then in 2.5% of 37% formaldehyde and slides were prepared similar to DCA. Slides were stained immediately before scoring with 50 &mu;g/ml acridine orange. Details of the procedure can be found in McNamee et al. (2009). Manual scoring was performed by all laboratories under fluorescent microscopy under 630 &times; magnification. The number of MN in 200 BNC was scored according to the criteria of Fenech et al. (2003).

### Dose estimates

Dose estimates were made based on each laboratory&apos;s own calibration curves for the appropriate assay. The dose-response curve from each laboratory was constructed using the conventional weighted Poisson regression model, Y &equals; c &plus; &beta;D&plus;&alpha;D^2^, where Y is the number of dicentrics/number of metaphase spreads scored or the number of MN/BNC, c is the background value, D is the radiation dose in Gray (Gy); and &beta; and &alpha; are dose and dose-squared coefficients used to estimate the rate of dicentrics in metaphase spreads or the rate MN/BNC. Maximum likelihood estimation was used to estimate the parameters of the fitted curves using either CABAS (Deperas et al. 2007) or Dose Estimate (Ainsbury and Lloyd 2010).

### Intercomparison design

Intercomparisons have been conducted annually within the Canadian biodosimetry network for the past 6 years. Each year the design of the intercomparison varied slightly but in general, it involved 10&ndash;12 *ex vivo* irradiated blood samples being blinded and shipped to each of the participating laboratories. Each laboratory was asked to identify the dose delivered to each sample using CDCA, QuickScan DCA and CBMN depending on which methods had been established in their laboratories. For DCA, data was recorded after scoring 20 and 50 cells (or 30 dicentrics) except for the first year where data was only recorded after 50 cells or 30 dicentrics. For CBMN, 200 BNC were scored. The time to score each sample was also recorded. Each laboratory was requested to have as many scorers as possible score each sample. A summary of the participation is found in Table II. Although a laboratory may have participated in the intercomparison and is included in the table, their results were only included in the analysis if at least two scorers from their laboratory participated in the intercomparison.

**Table II.** Summary of exercise participation.

Year	#Samples	# Labs participating	# Labs performing each assay			# Scorers performing each assay		
			DCA	QuickScan	CBMN	DCA	QuickScan	CBMN
2007	10	4	4	2	3	15	9	11
2008	10	6	6	4	3	18	17	13
2009	12	4	4	4	0	14	14	0
2010	10	6	6	6	3	19	19	12
2011	10	6	6	6	5	17	17	15
2012	10	5	5	5	4	13	13	11

DCA, dicentric chromosome assay; CBMN, cytokinesis block micronucleus.

### Statistical analysis

Analysis was performed to evaluate laboratory performance, dose estimates between methods in general and scoring methods between laboratories. The dose estimation was first assessed for consistency between laboratories within method of scoring and number of cells scored in order to compare the performance of the laboratories. Secondly, performance statistics were evaluated to compare dose estimates from the various methods and number of cells scored to the physical dose of radiation (reference value). Thirdly, the methods of scoring were compared between laboratories. In addition, dose estimates were compared to the actual dose and considered correct when within 0.5 Gy of the actual dose. Slides made from samples exposed to different doses of radiation were analyzed independently, and these are referred to as &lsquo;SampleID&rsquo;.

### Dose estimation

As previously mentioned, after slides were scored, each scorer estimated a dose of exposure based on their laboratory&apos;s calibration curve. Scorers only scored one replicate for each method of scoring and SampleID. Due to the limited number of replicates, the analysis to compare dose estimates is restricted at the laboratory level. Estimates of dose based on the calibration curves are assumed to be asymptotically normally distributed (Casella and Berger 2002). For this reason only laboratories with a minimum of two scorers were included in the analysis for comparing dose estimation results between laboratories or methods. Furthermore each year was analyzed separately.

An analysis of variance (ANOVA) model was used to assess differences in dose estimation between and within each method of scoring and number of cells scored. The assumptions for ANOVA (residuals are normally distributed, with constant variance between groups) were verified for each ANOVA model using Anderson-Darling test for normality and Levene&apos;s test for homogeneity. When the assumptions were not satisfied for the original scale of the data, then non-parametric Kruskal-Wallis test was applied. If the results were similar for the parametric and non-parametric approaches then the results based on the parametric approaches (assuming normality and constant variance across groups) were followed, indicating that the assumptions were adequately satisfied (Montgomery 2000). Pair-wise comparisons were conducted when the laboratory effect was significant (*p* &lt; 0.05), using Tukey&apos;s multiple comparison tests in order to control the overall Type I error to be less than 0.05.

### Performance statistics z and u for dose estimations (at the laboratory level)

The performance statistic z was applied to the dose estimation data to measure the deviation of each laboratory&apos;s estimated dose from the robust average. The robust average was determined using Algorithm A from ISO 5725-5:1998 (ISO 1998) which is currently suggested for proficiency testing to minimize the influence of outliers.

The performance statistics z is described here for comparing laboratories. For each laboratory, a z value was calculated using Equation (1):


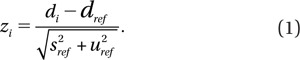


where *d_i_* is the reported estimated dose from the *i^th^* laboratory, and *d_ref_*, and *s_ref_* are the robust average and standard deviation (SD), respectively, as obtained from Algorithm A. The reference group from which *d_ref_* and *s_ref_* are evaluated is based on the CDCA method after scoring 50 cells. When the physical dose of radiation is known, then this is taken to be the reference value *d_ref_*. The term *u_ref_* is the standard uncertainty of *d_ref_* and is calculated as:


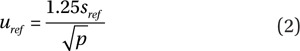


where, *p* is the number of participating laboratories. The value *u_ref_* is the uncertainty on the physical dose delivered. For each year of analysis, *u_ref_* was considered as negligible if the following criterion was satisfied (Equation 3):


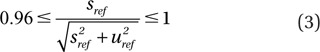


To evaluate laboratory performance, z statistics were interpreted as follows: |*z*| values &le; 2 were considered to be satisfactory, between 2 and 3 &lsquo;questionable&rsquo; and &ge; 3 &lsquo;unsatisfactory&rsquo;.

## Results

**Figure 1.**
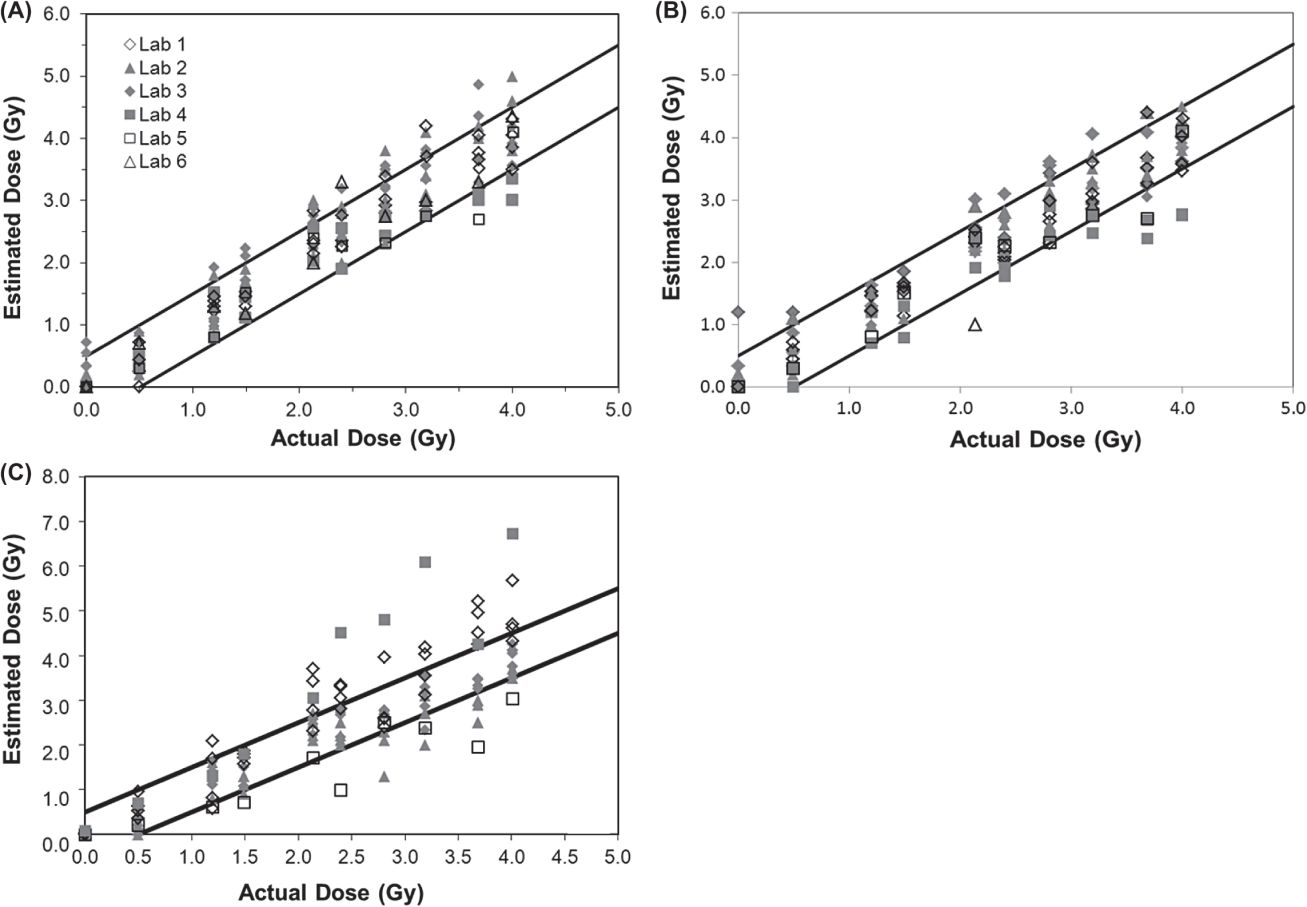
Intercomparisons across laboratories of estimated doses obtained using (A) conventional dicentric chromosome assay (CDCA) analysis of 50 metaphase spreads, (B) QuickScan DCA of 50 metaphase spreads, and (C) cytokinesis block micronucleus (CBMN) analysis of 200 binucleated cells. Each data point is a dose estimate from one individual, with scorers from each laboratory shown using the same symbol. The solid lines represent &plusmn; 0.5 Gy intervals.

Typical results from an intercomparison from a single year are shown in Figure 1(A&ndash;C) for CDCA, QuickScan DCA and CBMN assays. These figures show the results after scoring 50 cells for DCA and 200 cells for CBMN. Similar results after scoring 20 cells for DCA are not shown. The black solid lines represent the &plusmn; 0.5 Gy range from the actual dose delivered. Data from each laboratory is represented by a different symbol. The data from multiple years has been analyzed to provide an overview of the results from several exercises for the purposes of assessing biological trends. Examples of the statistical analysis are shown with a summary of the data in bar chart format below.

### Comparison of laboratories within each method of scoring and number of cells scored

**Figure 2.**
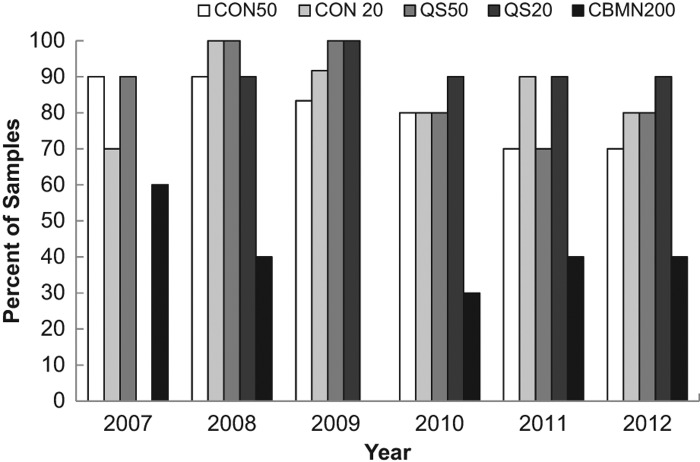
Illustration of the percentage of samples with dose estimates in agreement between laboratories for each year for each endpoint. A tally was prepared of the samples in which all laboratories were in agreement of the dose estimate (*p* &gt; 0.05). The average dose estimate from each laboratory and method was calculated and compared using ANOVA.

The first analysis was a comparison of the agreement in the dose estimate from each laboratory within each method and for each number of cells scored. Sample results are shown in Table III for sample 2 from 2008. For each sample from each year, the average dose estimate from each laboratory and method was calculated and compared using ANOVA. In order to generate Figure 2, a tally was made of the samples in which there was insufficient evidence to reject the null hypothesis in all laboratories (*p* &gt; 0.05). As demonstrated in Table III, the dose estimate from all laboratories agreed within each method except for the CBMN assay. In this case, the dose estimate from Laboratory 1 was statistically greater than that from Laboratory 2. Where data is missing, either that laboratory did not perform the assay or only one scorer analyzed the sample with that method.

**Table III.** Sample ANOVA analysis of data from year 2008; dose delivered &equals; 1.8 Gy.

Lab^a^	*n^b^*	Conventional method				QuickScan method				CBMN
		50 cells		20 cells		50 cells		20 cells		200 cells
		*d &plusmn; SD^c^*	*n*	*d &plusmn; SD*	*n*	*d &plusmn; SD*	*n*	*d &plusmn; SD*	*n*	*d &plusmn; SD*
1	4	2.05 &plusmn; 0.10	4	2.18 &plusmn; 0.38	4	2.03 &plusmn; 0.53	4	2.03 &plusmn; 0.53	4	2.78 &plusmn; 0.52
2	2	1.50 &plusmn; 0.71	2	1.80 &plusmn; 0.42	2	2.35 &plusmn; 0.35	2	2.35 &plusmn; 0.35	2	1.30 &plusmn; 0.71
3	6	2.00 &plusmn; 0.20	6	1.95 &plusmn; 0.23	7	1.97 &plusmn; 0.60	7	1.84 &plusmn; 0.71	7	2.16 &plusmn; 0.27
4	2	2.05 &plusmn; 0.21	2	2.20 &plusmn; 0.42	2	1.55 &plusmn; 0.21	2	1.35 &plusmn; 0.49		&ndash;
5	2	2.35 &plusmn; 0.21	2	2.40 &plusmn; 0.28	2	1.60 &plusmn; 0.57	2	1.00 &plusmn; 1.41		&ndash;
6	2	2.40 &plusmn; 0.28	2	1.95 &plusmn; 0.07		&ndash;		&ndash;		&ndash;
*^d^F* (*p*-value)		2.79 (0.0678)		1.18 (0.3735)		0.77 (0.5623)		1.21 (0.3574)		8.46 (0.0071)
										Lab 1 &gt; Lab 2 (*p* &equals; 0.0057)

CBMN, cytokinesis block micronucleus. ^*a*^Labs having less than two observations for a specific method by cell count were not included in the analysis. *^b^n* is the number of scorers in the lab that participated in the dose estimation exercise for the method. *^c^d &plusmn; SD* represents the average dose and standard deviation from the lab from all scorers. Where only one scorer from the lab participated then only that scorer&rsquo;s dose estimate is reported. ^*d*^The row F test is testing the null hypothesis of no difference in dose estimates between the different labs within a method and cell count.

**Figure 3.**
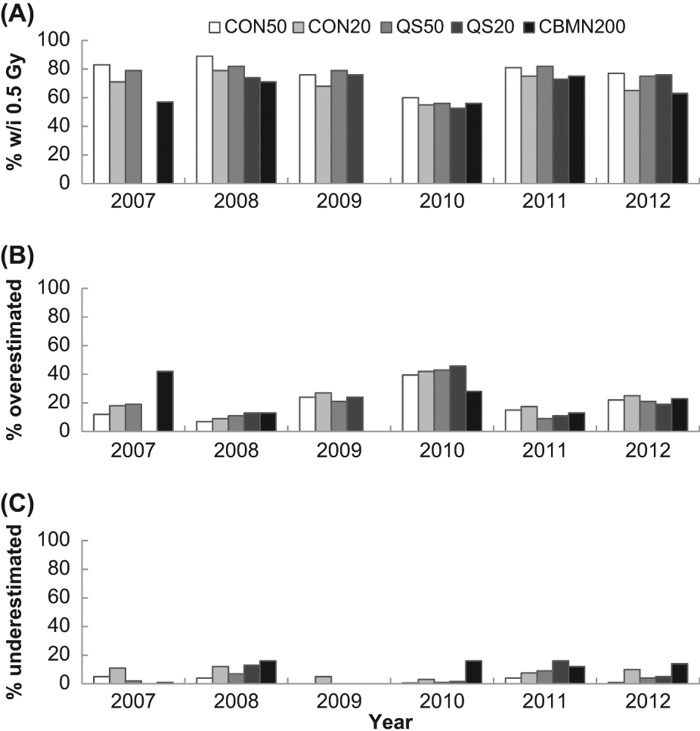
Illustration of the percentage of samples with dose estimates (A) within 0.5 Gy of the given dose, (B) more than 0.5 Gy over the given dose, and (C) more than 0.5 Gy under the given dose.

Figure 2 shows the agreement between laboratories for each year for each endpoint. For all assays except the CBMN assay, dose estimates from all laboratories were in agreement for more than 60% of the samples and in 19 out of these 22 cases, agreement occurred in 80% or more of the cases. This can be compared to a similar analysis based on the percent of correct dose estimates as those being within 0.5 Gy of the dose delivered to the sample (Figure 3) which also shows the percentage of samples over and underestimated. Similarly, both variations of the DCA performed better than the CBMN, however, based on this criteria, the CBMN assay was consistently correct at least 55% of the time in all years tested. Figure 3 also demonstrates that a greater number of samples were overestimated rather than underestimated.

**Figure 4.**
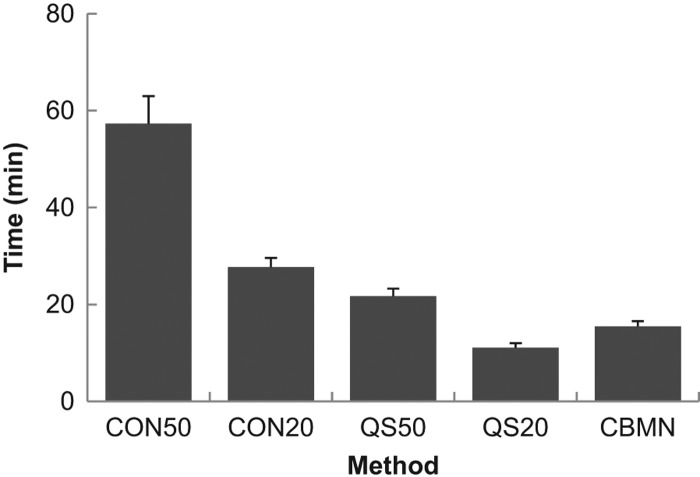
Illustration of the average time required to score one sample for each method.

Figure 4 illustrates the time it took to score one sample averaged over all scorers and all years except 2007 when no times were recorded. It is evident that scoring 50 cells by CDCA scoring was the most time-consuming, requiring almost 1 h to score a single sample. The time to score decreased for all of the other endpoints, with QuickScan scoring of 20 cells being the quickest, requiring about 10 min per sample.

### Performance statistics z for dose estimations

Performance statistic z for the dose estimates was calculated using the physical dose of radiation as the reference value for the robust average (d*ref*). The reference standard deviation (s*ref*) was based on the CDCA method after scoring 50 cells. The objective was to define a reference interval to allow comparison of the effectiveness of the QuickScan and CBMN method with respect to the CDCA method, as well as compare the effectiveness of scoring 20 versus 50 cells (or 200 cells in the CBMN method). Values of |z| &le; 2 were considered to be satisfactory. Sample data from this analysis is presented in Table IV.

**Table IV.** Performance statistics z sample analysis of data from year 2008; dose delivered &equals; 1.8 Gy.

	Conventional method				QuickScan method				CBMN	
	50 cells		20 cells		50 cells		20 cells		200 cells	
Lab^*a*^	*d&plusmn; SD^b^*	*z^c^*	*d&plusmn; SD*	*z*	*d&plusmn; SD*	*z*	*d&plusmn; SD*	*z*	*d&plusmn; SD*	*z*
1	2.05 &plusmn; 0.10	0.64	2.18 &plusmn; 0.38	0.96	2.03 &plusmn; 0.53	0.53	2.03 &plusmn; 0.53	0.58	2.78 &plusmn; 0.52	2.5
2	1.50 &plusmn; 0.71	&minus; 0.77	1.80 &plusmn; 0.42	0.00	2.35 &plusmn; 0.35	1.41	2.35 &plusmn; 0.35	1.41	1.30 &plusmn; 0.71	&minus; 1.28
3	2.00 &plusmn; 0.20	0.51	1.95 &plusmn; 0.23	0.39	1.97 &plusmn; 0.60	0.44	1.84 &plusmn; 0.71	0.11	2.16 &plusmn; 0.27	0.92
4	2.05 &plusmn; 0.21	0.64	2.20 &plusmn; 0.42	1.03	1.55 &plusmn; 0.21	&minus; 0.64	1.35 &plusmn; 0.49	&minus; 1.16		
5	2.35 &plusmn; 0.21	1.41	2.40 &plusmn; 0.28	0.28	1.60 &plusmn; 0.57	&minus; 0.51	1.00 &plusmn; 1.41	&minus; 2.05		
6	2.40 &plusmn; 0.28	1.54	1.95 &plusmn; 0.07	0.39						

CBMN, cytokinesis block micronucleus; ^*a*^Labs having less than 2 observations for a specific method by cell count were not included in the analysis. ^*b*^d &plusmn; SD represents the average dose and standard deviation from the lab from all scorers. Where only one scorer from the lab participated then only that scorer&rsquo;s dose estimate is reported. ^*c*^z-scores were calculated using the dose delivered (1.8 Gy) and the robust standard deviation (*s*&ast;) obtained from the conventional method at 50 cells, across all labs having greater than 1 observation. The robust average and robust standard deviation were (*x*&ast; &equals; 2.07, s* &equals; 0.35), both obtained from the conventional method at 50 cells, across all labs having greater than 1 observation.

**Figure 5.**
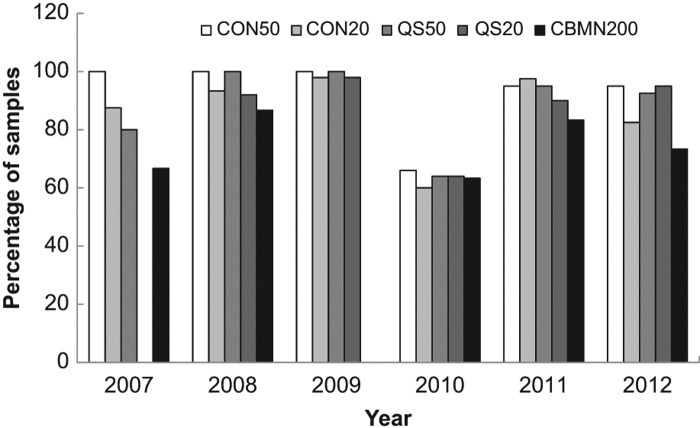
A comparison of the percentage of correctly evaluated samples based on a |z| &le; 2 for each method across all years of the intercomparisons.

Similar to above, the data from this analysis was tallied to get a better overall view of the results. Samples with values of |z| &le; 2 were tallied as being satisfactorily close to the physical dose. Figure 5 shows the tallied data for all laboratories and all methods as a function of intercomparison year. If the data from 2010 is excluded, it can be seen that scoring 50 cells using CDCA (CON50) was extremely successful (98%) and scoring 20 cells using CDCA (CON20) was in agreement with the physical dose 90% of the time. QuickScan was also in agreement with the physical dose at least 90% of the time after scoring either 20 or 50 cells. Only the CBMN assay fell below 80% agreement in two of the intercomparison years.

**Figure 6.**
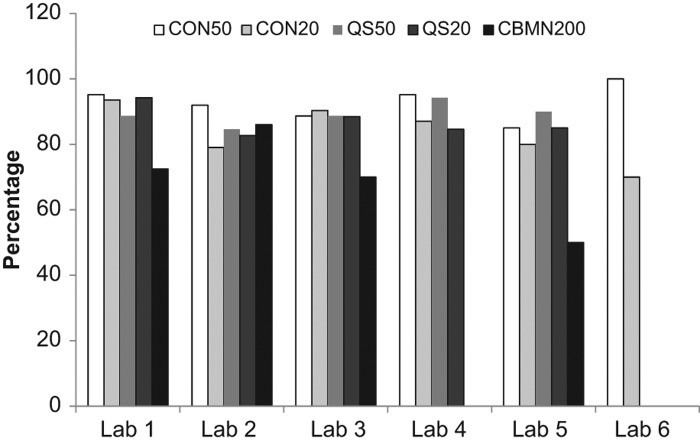
A comparison of the percentage of correctly evaluated samples based on a |z| &le; 2 for each method in each laboratory.

**Figure 7.**
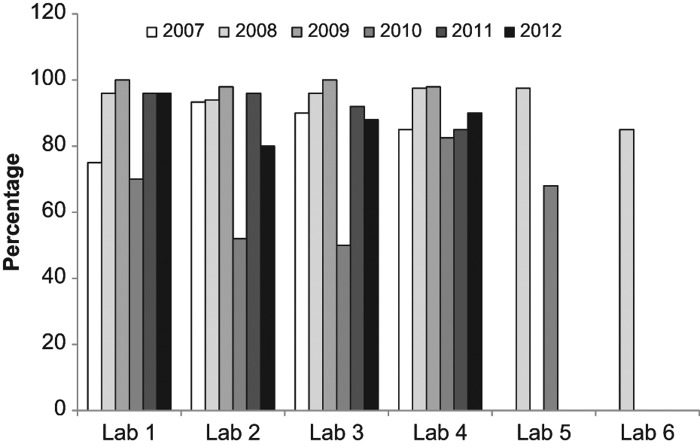
A comparison of the percentage of correctly evaluated samples based on a |z| &le; 2 for each year in each laboratory.

In 2010, the data reflects a change in the exposure conditions that was not matched with the appropriate change in calibration curves. This data set was also separated by laboratory to examine how each laboratory performed each method and how their performance changed year to year. Figure 6 shows the analysis of how each laboratory performed for each method averaged over all years. Similarly to Figure 5, most endpoints resulted in dose estimates similar to the physical dose except in the case of the CBMN assay that had as low as 60% agreement. The data is also presented for each laboratory based on yearly performance over all endpoints (Figure 7). In general, each laboratory&apos;s performance was maintained from year to year (except 2010) with some small fluctuations. Data is missing for Laboratories 5 and 6 due to only one scorer performing the analysis or that laboratory not participating in those years.

## Discussion

The Canadian NBDRP was conceived in 2002 and by 2005 became well established as a network comprised of four reference laboratories in addition to 18 satellite laboratories in existing hospital genetics departments who had been trained to provide surge capacity for the scoring of dicentrics (Miller et al. 2007). By 2007, a program of annual intercomparisons to maintain the expertise and confirm the capabilities and capacity of the network had been established. In 2008, two laboratories in the United States joined the network to create a North American Biodosimetry Network and periodically participated in the Canadian annual intercomparisons. This paper summarizes these outcomes and describes the importance of performing such intercomparisons.

Overall, the results of these intercomparisons demonstrate the success of the NBDRP in terms of capabilities, capacities and accuracy in dose estimates being well maintained from year to year. The number of participating scorers fluctuated, depending mostly on whether or not the US laboratories took part in the intercomparison. There was some minor fluctuation within the Canadian laboratories which was due to expected changes in staffing. In 2010, the exposure conditions were not well matched to the calibration curves at each laboratory resulting in a large number of overestimated doses. This highlights the importance of having the appropriate calibration curves for intercomparisons in order to effectively assess the capabilities of the participating laboratories. However, even with poorly matched calibration curves, samples that were exposed were clearly identified and the results would be sufficient for mass casualty events.

These intercomparisons provided an opportunity to compare the agreement in dose estimates between each laboratory based on scoring methods, numbers of cells scored and assays used. It was clear that, of all the assays performed, the CBMN assay had the lowest agreement between laboratories. Although there was agreement only 30&ndash;60% of the time with the CBMN based on the ANOVA models (comparing dose estimates between laboratories), when the percent of satisfactory dose estimates was determined by laboratory based on z-score analysis (comparing dose estimates to the physical dose), the CBMN assay was satisfactory at least 60% and usually over 80% of the time. There are several reasons for the reduced performance of the CBMN assay including greater inter-individual variation in the sample donor background levels and response to ionizing radiation. These can lead to larger variations in the calibration curves between laboratories which will result in an increased range of dose estimates reported (Fenech 2010). The results also demonstrated that the QuickScan DCA analysis, when 50 cells were scored, was as accurate, if not better than CDCA scoring based on agreement in dose estimates between laboratories. When compared to conventional scoring of 50 cells, QuickScan scoring of 50 cells produced accurate dose estimates for most samples. Even when only 20 cells were enumerated with either conventional or QuickScan scoring, satisfactory dose estimates were made, on average, in over 90% of the samples. In addition, when scoring time of the samples is taken into account, QuickScan scoring of 20 cells required on average 10 min per sample as compared to nearly 30 min per sample for the conventional scoring of the same number of cells and about 15 min for CBMN scoring of 200 cells. In summary, these results indicate that for a mass casualty situation, scoring 20 cells using QuickScan would provide an excellent triage dose estimation method.

The fluctuation in results from year to year emphasizes the importance of annual intercomparisons. These fluctuations are likely due to changes in staff, technical experience with the assays and amount of biodosimetry a laboratory performed between intercomparisons. The laboratory with the most consistent results from year to year (laboratory 3) was also the laboratory with the smallest turnover in staff and the longest history of performing biological dosimetry. Laboratory 1 also had a small turnover in staff but less experience before the formation of the network. These observations indicate that for laboratories that do not perform biodosimetry on a regular basis, annual testing provides an opportunity to assess their capabilities and practice their skills. Also, when new staff is hired, it can be used to ensure adequate training has been performed.

These intercomparisons are also an excellent opportunity to identify gaps in the processes required to receive and analyze multiple samples on short notice. They have helped the Network draft and revise standard operating procedures as &lsquo;lessons learned&rsquo; were identified and addressed each year. For example, many issues with shipping arose with each intercomparison which has resulted in well- defined procedures and protocols for packaging, labelling and shipping biological samples by air, both within Canada and internationally.

## Conclusion

Maintenance of a network of laboratories for emergency response biodosimetry needs to be continuous and rigorous in order to have confidence in the dose estimates being generated. One way to maintain the capacity and capability of a network is through annual intercomparisons which evaluate the abilities and expertise within the network and allow for an opportunity to test protocols and practice procedures. This type of intercomparison is necessary to maintain confidence that the network is in a state of readiness for emergency response. This study has demonstrated, through 6 years of intercomparisons, that the Canadian biodosimetry network is capable of producing dose estimations over a variety of different assays quickly and accurately. These findings provide confidence to the medical community, the public and government bodies that in the event of a nuclear accident, biodosimetry can be applied to manage and medically treat casualties to ensure the minimization of health risks.
